# Oxidative protein biogenesis and redox regulation in the mitochondrial intermembrane space

**DOI:** 10.1007/s00441-016-2488-5

**Published:** 2016-09-08

**Authors:** Phanee Manganas, Lisa MacPherson, Kostas Tokatlidis

**Affiliations:** Institute of Molecular, Cell and Systems Biology, College of Medical, Veterinary and Life Sciences, University of Glasgow, Glasgow, UK

**Keywords:** Mitochondria, Protein import, Intermembrane space, Redox regulation, Oxidative folding

## Abstract

Mitochondria are organelles that play a central role in cellular metabolism, as they are responsible for processes such as iron/sulfur cluster biogenesis, respiration and apoptosis. Here, we describe briefly the various protein import pathways for sorting of mitochondrial proteins into the different subcompartments, with an emphasis on the targeting to the intermembrane space. The discovery of a dedicated redox-controlled pathway in the intermembrane space that links protein import to oxidative protein folding raises important questions on the redox regulation of this process. We discuss the salient features of redox regulation in the intermembrane space and how such mechanisms may be linked to the more general redox homeostasis balance that is crucial not only for normal cell physiology but also for cellular dysfunction.

## Introduction

**Fig. 1:**
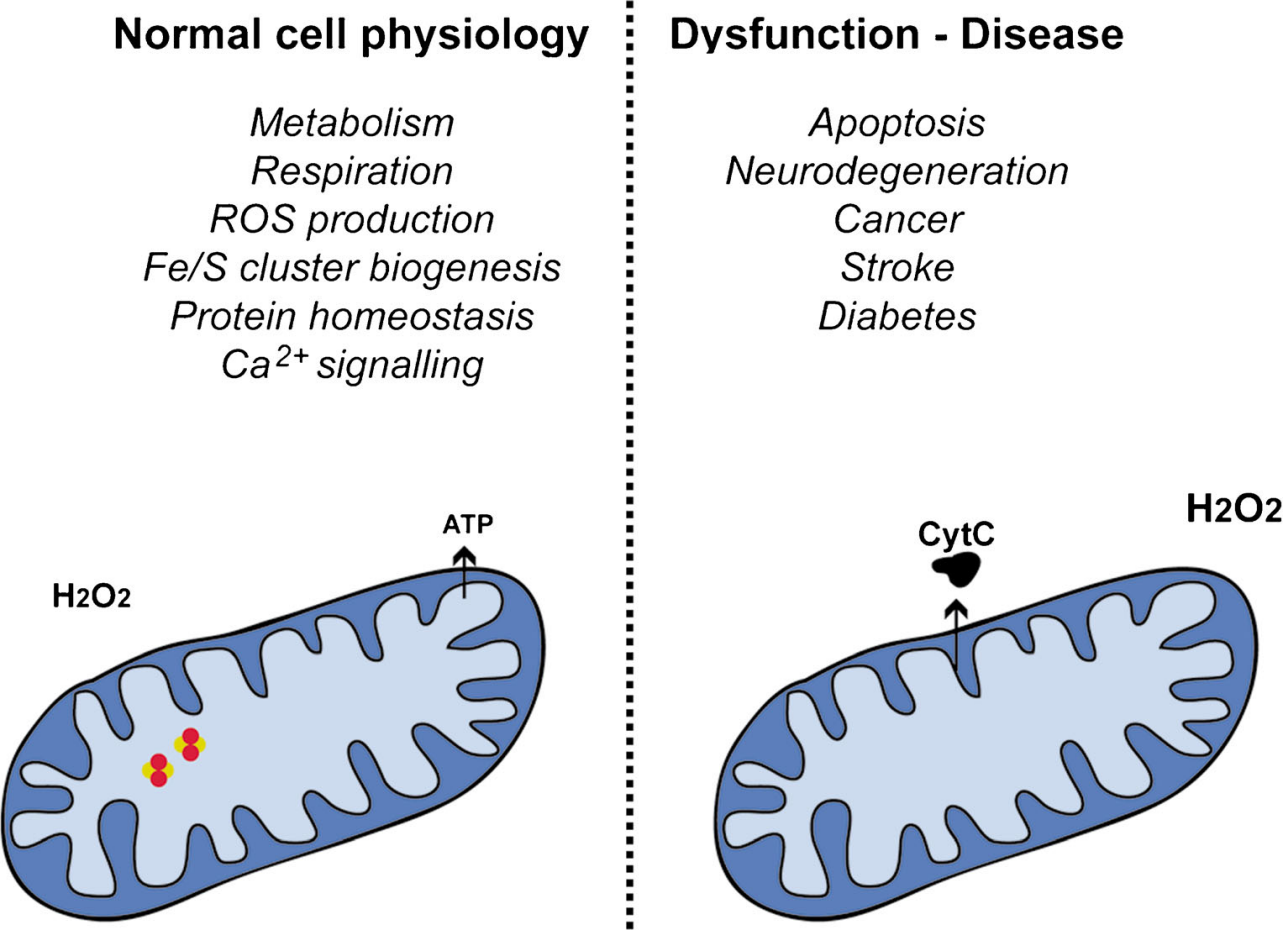
Mitochondria are involved in a series of different cellular processes. These include physiological cellular functions, such as respiration and metabolic regulation, essential chemical processes, such as iron/sulfur cluster biogenesis and oxidative folding, as well as signalling mechanisms involving molecules such as calcium and reactive oxygen species. Mitochondria also play an important role during disease and cellular dysfunction and are responsible for the initiation of apoptosis. The figure is a schematic of mitochondrial structure and is not drawn to scale

Mitochondria are subcellular organelles with a distinct structure and are involved in a variety of cellular processes, which include, but are not limited to, energy production, apoptosis and iron/sulfur cluster assembly (Fig. 1). These organelles are characterised by the presence of two membranes of different composition: the outer membrane (OM) and the inner membrane (IM). The presence of these two membranes allows the formation of two aqueous subcompartments within the mitochondria, which are the intermembrane space (IMS) and the matrix. Each one of these compartments is characterised by a specific set of proteins that carry out specialised functions. The majority of these proteins are encoded in the nuclear genome and synthesised in the cytosol, making it necessary for the mitochondria to possess mechanisms through which to import all the proteins required for the correct function of the organelle (Neupert 1997). During the import process, the incoming proteins are targeted to their correct location within the organelle by utilising a series of different import pathways.

## General import pathways

**Fig. 2:**
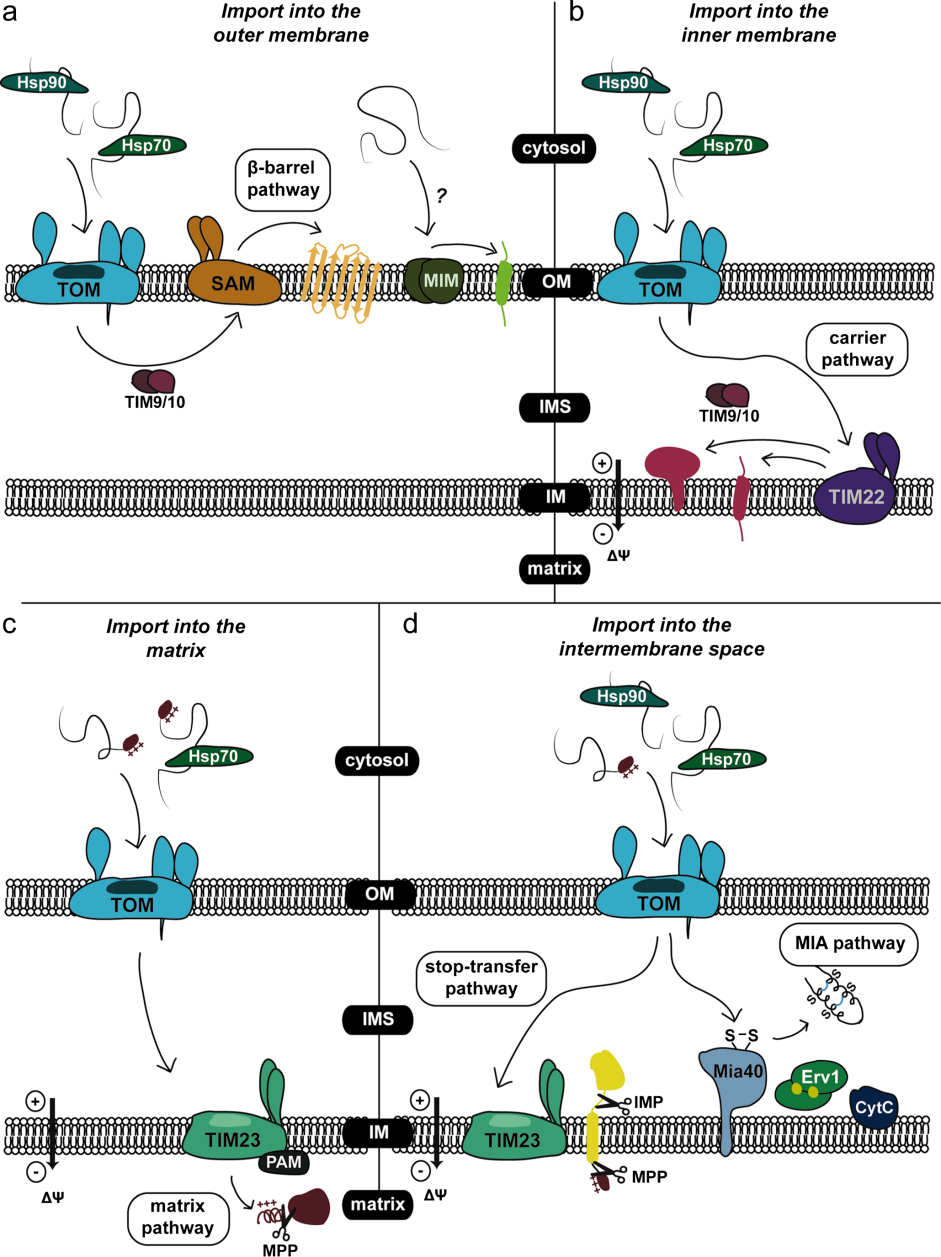
Mitochondrial import pathways. Incoming proteins interact with cytosolic chaperones (Hsp70/Hsp90) and enter the mitochondria through the general entry gate, the translocase of the outer membrane (*TOM*) complex. **a** Protein import into the outer membrane of mitochondria. Once the precursors are localised in the intermembrane space (*IMS*), they interact with the mitochondrial chaperone translocase of the inner membrane (*TIM9/10*) complex and are targeted to the sorting and assembly machinery (*SAM*) complex for insertion into the outer membrane. This pathway is followed by β-barrel proteins. The less well-studied mitochondrial import pathway (*MIM*) may be responsible for the insertion of single- or multi-spanning α-helical outer membrane proteins, in a mechanism that remains unknown. **b** Protein import into the inner membrane of mitochondria. In the *IMS*, the precursors interact with the mitochondrial chaperone (*TIM22*) complex and are inserted into the inner membrane. **c** Protein import into the matrix. Proteins that are destined to the innermost compartment of mitochondria follow the mitochondrial chaperone (*TIM23*) pathway. The presence of a positively charged N-terminal MTS guides the protein through the *TIM23* complex, with the translocation being facilitated by the presequence translocase-associated motor (*PAM*) complex. After the protein has been imported into the matrix, the mitochondrial processing peptidase (*MPP*) cleaves the MTS and the mature protein is released. **d** Protein import into the mitochondrial intermembrane space. In the *IMS*, proteins that contain bipartite presequences follow a variation of the *TIM23* pathway known as a “stop-transfer”. The precursors are partially translocated into the matrix and become arrested at the *TIM23* pore due to the presence of a hydrophobic region. Through the action of the *MPP* and the inner membrane protease (*IMP*), the protein is released into the *IMS*. Proteins that contain cysteine residues interact with the oxidoreductase Mia40, which is responsible for the introduction of disulfide bonds, therefore trapping them in the *IMS*

The majority of mitochondrial preproteins are encoded in the nucleus and translated in the cytosol, before being imported into mitochondria (Neupert 1997). In order for the import process to be more efficient, these precursor proteins are maintained in an unfolded state in the cytosol, through association with a series of different chaperones. The targeting of mitochondrial precursor proteins is influenced by targeting sequences found within the preprotein. This is typically an N-terminal presequence—or, matrix targeting sequence (MTS)—which will target the preprotein to the matrix unless it also contains further targeting information. The MTS is normally an amphipathic α-helix with positive charges on the one side of the helix and hydrophobic residues on the other. This presequence is usually (but not always) cleaved after import through the function of the mitochondrial-processing peptidase (MPP) (Braun and Schmitz 1997). Many mitochondrial preproteins additionally contain an internal targeting sequence, which can affect the route of import of the precursor and in which mitochondrial compartment it will eventually end up. The two main general import translocases of mitochondria are the translocase of the outer membrane (TOM) complex of the outer membrane and the translocase of the inner membrane (TIM23) complex of the inner membrane (Fig. 2)

### Outer membrane complexes

The TOM (translocase of the outer membrane) complex is the general route of entry for all mitochondrial precursor proteins (Fig. 2a). It aids in the release of binding cytosolic factors from the preproteins as well as their correct folding upon entry into the IMS. It has seven components, which can be split into two main groups. Tom20, Tom70 and Tom22 make up the receptors that interact with cytosolic substrate proteins, with Tom20 and Tom70 being the main receptors. Each one of these two proteins has a distinct preference for certain substrate proteins, but they are also able to compensate one another’s function to a certain extent (Neupert and Herrmann 2007). Both are anchored to the outer membrane by their N-terminal domains, thus exposing hydrophilic domains to the cytosol where they can interact with the incoming substrates. Tom20 contains a binding groove for the hydrophobic residues of the MTS (Abe et al. 2000), whereas Tom70 recognises the internal targeting sequences of preproteins (Chan et al. 2006). Tom22 differs from the other two in that it exposes a negatively charged N-terminus to the cytosol, and its C-terminus to the IMS (van Wilpe et al. 1999). The other main group of TOM complex components are the ones that make up the pore. Tom40 is the central component of the pore and contains a binding region for mitochondrial preproteins (Neupert and Herrmann 2007; Shiota et al. 2015). Tom5, Tom6 and Tom7 are also part of the pore, though non-essential for the function of the TOM complex unless all three subunits are deleted (Dietmeier et al. 1997; Dekker et al. 1998; Sherman et al. 2005).

The TOM complex is also involved in the insertion of proteins into the outer membrane of mitochondria. However, this process requires other membrane complexes, such as the sorting and assembly machinery (SAM) complex for β-barrel proteins (Fig. 2a). Mitochondria and chloroplasts are the only eukaryotic organelles which contain these β-barrel proteins—porin and Tom40, for example—most likely due to their shared lineage from prokaryotic cells (Neupert and Herrmann 2007). Sam50 is the main component of the SAM complex and is highly conserved. It has two domains: an IMS-exposed N-terminal region which is hydrophilic, and a C-terminal domain which forms its β-barrel structure. Sam35 and Sam37 are two other subunits which make up the structure, though only Sam50 and Sam35 are essential for viability (Wiedemann et al. 2003; Chan and Lithgow 2008). Outer membrane protein precursors interact with the TOM complex and move through its pore to the IMS. Small Tims are then able to bind to these preproteins and guide them to the SAM complex, through which they are inserted into the outer membrane (Höhr et al. 2015).

One last, rather distinct, import pathway of the outer mitochondrial membrane is the MIM (mitochondrial import) complex involving the mitochondrial import protein 1 (Mim1). This protein was found to be important in the import of both single- and multi-spanning α-helical outer membrane proteins and facilitates their more efficient integration into the outer membrane (Fig. 2a) (Becker et al. 2008, 2011; Popov-Celeketić et al. 2008; Papic et al. 2011). More recently, the protein Mim2 was also found to be a component of this particular complex and absence of this protein leads to impaired mitochondrial protein import, morphological defects in mitochondria, while also creating problems in the correct assembly of the TOM complex (Dimmer et al. 2012; Neupert 2015).

### Inner membrane complexes

The import of inner membrane proteins, such as the solute carrier family and membrane-embedded Tims (Tim17, Tim22 and Tim23), relies on the TIM22 pathway (Fig. 2b) (Neupert and Herrmann 2007). The substrates of this pathway share structural similarities in that they all expose both their N- and C-termini to the IMS. The TIM22 pathway relies on three protein complexes: the TOM complex, small TIM complexes and the TIM22 translocase itself. Small Tims, i.e. Tim8, Tim9, Tim10, Tim12 and Tim13, contain twin CX3C motifs, and form hetero-oligomeric complexes which are soluble in the IMS and associate with the TIM22 complex (Kovermann et al. 2002; Vergnolle et al. 2005). Of these, Tim9, Tim10 and Tim12 are encoded by essential genes in *Saccharomyces cerevisiae* and are involved in the recognition of substrates. The TIM22 complex itself is made up of Tim22, Tim54 and Tim18. Tim22 forms the core of the complex, and is homologous to both Tim23 and Tim17 of the TIM23 complex (Sirrenberg et al. 1996). Tim54 and Tim18 are accessory proteins, with Tim54 being non-essential (Kerscher et al. 1997; Kovermann et al. 2002).

At the beginning of the TIM22 import pathway, the cytosolic chaperone Hsp70 guides the carrier protein precursors to the receptors of the TOM complex (Komiya et al. 1997). These preproteins pass though the TOM complex and form translocation intermediates interacting with the Tim9–Tim10 complex in the IMS. This small TIM complex protects the hydrophobic regions of the preprotein to prevent its aggregation in the IMS (Truscott et al. 2002; Koehler 2004; Webb et al. 2006). The carrier protein precursors are then delivered to the TIM22 complex and inserted into the inner membrane in a reaction that is dependent on the inner membrane potential, where they are able to form dynamic dimers (Dyall et al. 2003). Tim23 import is similar to the import of carrier proteins, but uses the non-essential Tim8–Tim13 complex to chaperone the preprotein in the IMS instead (Paschen et al. 2000).

TIM23 (translocase of the inner membrane) facilitates the translocation of all matrix preproteins, as well as some that are destined for the inner membrane and the IMS (Fig. 2c). The TIM23-dependent matrix import pathway is powered both by the membrane potential of the inner membrane and ATP hydrolysis, and, under high oxidative metabolism activity, its substrates can compose up to 20 % of the total cellular proteins (Neupert and Herrmann 2007). The TIM23 complex has two main groups of components: the membrane channel and the import motor. Tim23 and Tim17 make up the core of the membrane channel complex, and both proteins expose N-terminal domains to the IMS (Donzeau et al. 2000). The N-terminal domain of Tim23 contains a coiled-coil domain for dimerisation and substrate binding (Bauer et al. 1996), and its N-terminus stretches to the outer membrane (Donzeau et al. 2000). Tim17, on the other hand, exposes a much shorter N-terminal domain with conserved, negative residues, and is thought to be involved in the gating of the channel (Meier et al. 2005). Tim50, another component of the channel, is anchored to the inner membrane by its N-terminus, and exposes a domain able to interact with preproteins into the IMS (Geissler et al. 2002). The final component of the channel, Tim21, is non-essential but interacts with the IMS domain of Tom22 (Chacinska et al. 2005; Mokranjac et al. 2005).

The PAM (presequence translocase-associated motor) complex is required for further import of preproteins into the matrix, after their N-terminal MTS has been transferred across the inner membrane by the membrane potential (Fig. 2c). Tim44 is the main component of the motor, and is a hydrophilic matrix protein attached to the inner membrane. It also contains a hydrophobic pocket to which substrates—guided by Hsp70—can bind (Josyula et al. 2006). Hsp70, the matrix chaperone protein, contains an N-terminal ATPase domain and C-terminal substrate-binding domain, and cycles between ADP- and ATP-bound states via the nucleotide exchange protein Mge1. The ATP-bound form is recruited by Tim44 into the import motor structure (Young et al. 2004; Bukau et al. 2006). Finally, Tim14 and Tim16 are DnaJ-like proteins that regulate the binding of substrates to Hsp70.

### IMS-specific import pathways

All proteins that are resident within the IMS are encoded by nuclear genes and become synthesised in the cytosol. As such, in order to reach their final destination, they can follow specific pathways that allow for their translocation across the outer mitochondrial membrane and their retention in the IMS. The two most well-characterised classes of IMS proteins are (1) proteins that contain bipartite presequences and (2) proteins that depend on Mia40 for their import.

Bipartite presequences are N-terminal targeting signals that consist of two distinct regions: an N-terminal mitochondrial targeting signal (MTS) followed by a hydrophobic region reminiscent of a transmembrane domain. This particular import pathway is known as the stop-transfer pathway (Fig. 2d), as the presence of the hydrophobic region stops the translocation of the protein through the inner mitochondrial membrane (Glick et al. 1992). Just like in the case of the TIM23 pathway, the MTS presequence is cleaved off by MPP. The next step involves the action of an intermembrane space protease (e.g. IMP or Pcp1), which will remove the hydrophobic sorting signal and lead to the release of the protein in the IMS (Nunnari et al. 1993; Esser et al. 2002; McQuibban et al. 2003). In this pathway, ATP hydrolysis is not required and the import of proteins can be powered solely by the membrane potential, which is sufficient to engage the preprotein in the TIM23 translocase.

A distinct pathway exists for IMS proteins that contain Cys residues. The existence of an oxidative folding pathway had been proposed based on the demonstration of the presence of internal disulfides *in vivo* for the small Tim proteins (Curran et al. 2002; Lu et al. 2004). The key component for this pathway, which is known as the MIA (mitochondrial import and assembly) pathway, is the Mia40 protein (Fig. 2d) (Chacinska et al. 2004; Naoé et al. 2004; Hell 2008). Mia40 is an oxidoreductase and acts as a disulfide donor protein for imported precursors. The TIM23 complex anchors Mia40 to the inner mitochondrial membrane by its N-terminus, and leaves its C-terminus exposed to the IMS, allowing Mia40 to interact with its substrates (Chatzi et al. 2013). Mia40 is responsible for the introduction of disulfide bonds to the preproteins, resulting in their folding and trapping within the IMS (Chacinska et al. 2004; Gabriel et al. 2007; Sideris and Tokatlidis 2010). The detailed molecular mechanism of this process will be described later in the text. Substrates for the MIA pathway contain either twin CX3C or CX9C motifs that associate with the hydrophobic binding cleft of Mia40. These substrates then interact with the conserved CPC motif of Mia40, in which the second cysteine residue forms a mixed disulfide intermediate with the substrate protein (Banci et al. 2009). As previously mentioned, CX3C proteins include the small Tims, which function as chaperone protein complexes aiding movement of membrane proteins through the IMS (Sirrenberg et al. 1996; Koehler et al. 1998). Other Mia40 substrates, such as the COX proteins which contain twin CXC9 motifs, are often involved in stabilising or assembling the mitochondrial respiratory chain (Herrmann and Hell 2005; Chatzi and Tokatlidis 2013). More recent work has shown that the import of certain proteins, such as Atp23, and Mrp10 into the IMS and Tim22 into the inner membrane, is dependent on Mia40, but this occurs through an interaction that does not require any of their cysteine motifs (Weckbecker et al. 2012; Wrobel et al. 2013; Longen et al. 2014).

Erv1, the second component of the MIA pathway, is a flavin adenine dinucleotide (FAD)-linked sulfhydryl oxidase. Erv1 is unique in that it shares no structural similarity to other Mia40 substrates (Chatzi and Tokatlidis 2013), but contains three conserved cysteine pairs (C30/C33, C130/C133 and C159/C176) (Hofhaus et al. 2003). The first cysteine pair acts as the shuttle disulfide interacting with Mia40 (Lionaki et al. 2010), and the third as a structural disulfide. The structural disulfide is the one recognised during the import process of Erv1 by Mia40 (Terziyska et al. 2009), whilst complete folding of Erv1 also requires FAD binding (Kallergi et al. 2012). Once properly folded, Erv1 has a critical role in the electron transfer process underpinning the oxidative folding pathway. In particular, the electrons are removed from Mia40 through the N-terminal redox active CX2C motif (distal or shuttle motif) and are transferred to the FAD-proximal CX2C motif of Erv1. From there, they are transferred onto the FAD molecule itself, which is responsible for the shuttling of the electrons either directly to molecular oxygen—in a reaction that leads to the production of hydrogen peroxide (H_2_O_2_)—or through cytochrome *c* and the respiratory chain to oxygen, a process which produces H_2_O (Farrell and Thorpe 2005; Ang and Lu 2009; Bien et al. 2010; Banci et al. 2011). There are alternative final electron acceptors in this process, namely the cytochrome *c* heme lyase Ccp1 (Dabir et al. 2007). Additionally, the process also operates under anaerobic conditions, but whether there are other proteins in addition to Mia40 and Erv1 involved in this case is not yet understood. Although Erv1 does not have the capacity to oxidise the substrates on its own, it has been proposed to function as part of a ternary complex together with Mia40 in vivo (Stojanovski et al. 2008). Further reconstitution studies with purified components will provide valuable insights into the detailed mechanism.

## Redox regulation

**Fig. 3:**
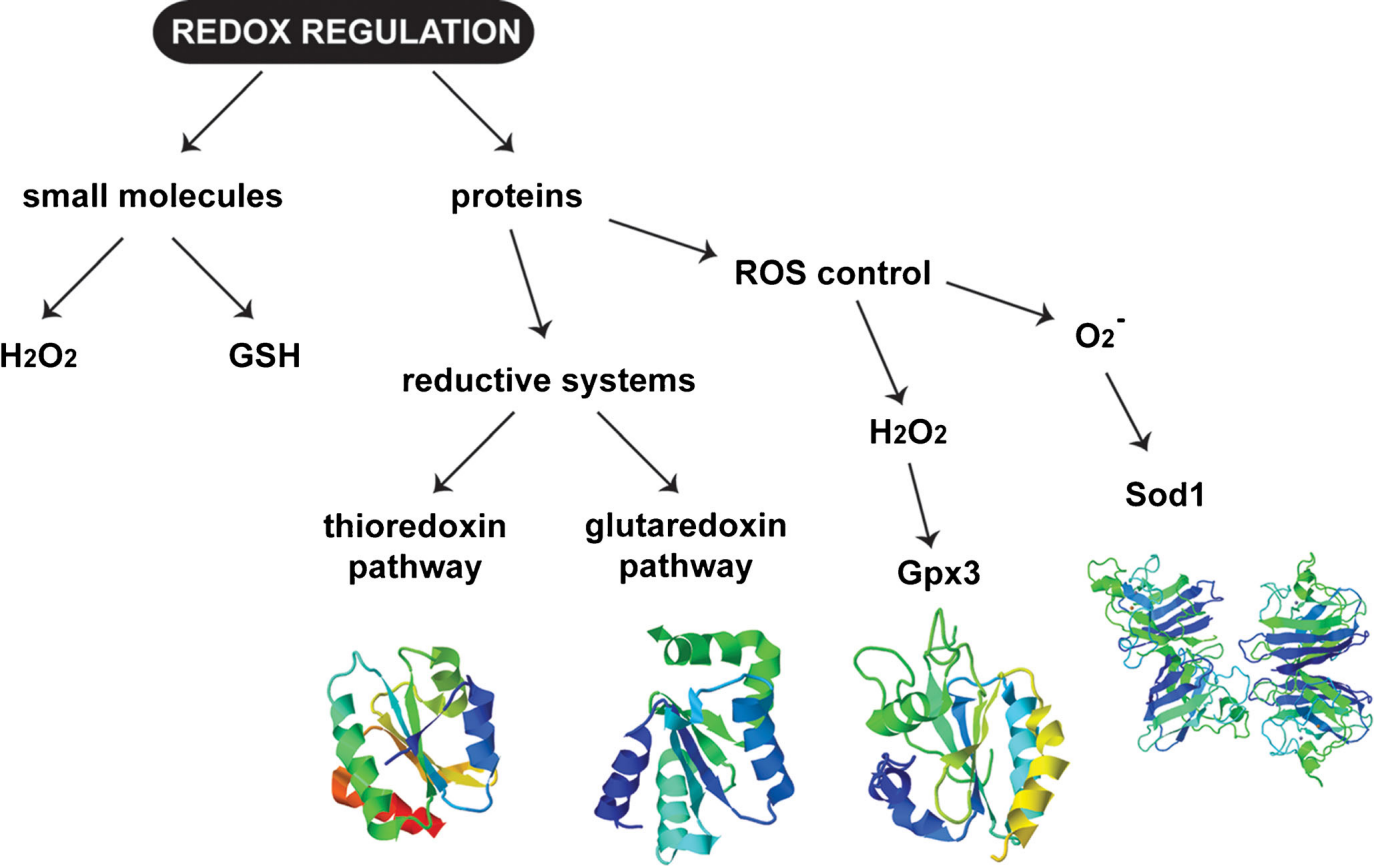
Cellular redox regulation. Cells have a series of different mechanisms to combat the effects of redox imbalance. These can be divided into two categories: small molecules, such as H_2_O_2_ and glutathione, which act like signals and are important for the initiation of the redox response; and proteins, which are able to detect alterations in the levels of reactive oxygen species (Gpx3, Sod1), the GSH:GSSG ratio (glutaredoxin system) as well as in the redox state of proteins (thioredoxin system). The structures shown were obtained from the PDB website (http://www.rcsb.org/pdb/home/home.do) and are the following: thioredoxin pathway—Trx1 (PDB code: 2N5A), glutaredoxin pathway—Grx2 (PDB code: 3CTF), Gpx3 (PDB code: 3CMI) and Sod1 (PDB code: 1SDY)

The MIA pathway stands out from the other import pathways in mitochondria as it is the only one that results in a chemical modification of the precursor through the formation of intramolecular disulfide bonds. This property raises the question of how the redox regulation circuitry that controls the cellular redox state is linked to the function of the MIA machinery. In more general terms, there have been a number of studies that suggest a role for small redox active molecules, such as H_2_O_2_ and glutathione (GSH), in the oxidative folding process (Fig. 3). These factors are an important part of the cellular redox homeostasis and can modulate oxidative stress conditions within the cellular environment.

All organisms are exposed to reactive oxygen species (ROS) as part of their normal growth cycle. These ROS, which include H_2_O_2_, the superoxide anion (O_2_^−^) and the hydroxyl radical (OH^−^), are either a result of physiological cellular processes or an effect caused by the exposure of cells to radical-generating compounds. The production of these molecules within the cell can lead to a series of effects, including, but not limited to, the modification of DNA, lipid peroxidation and protein oxidation (Morano et al. 2012). As such, oxidative stress frequently leads to cell death and can be the causative factor in the ageing process, as well as in a number of different diseases (Gutteridge and Halliwell 2000; Grant 2008).

In order for the cells to protect themselves from the damaging effects of ROS, they have developed a series of different mechanisms which are able to detoxify the cell and restore redox homeostasis. Such processes include (1) the transcriptional and translational regulation of genes encoding antioxidant enzymes, and (2) the post-translational modification of proteins involved in physiological cellular processes (Grant 2008).

A prime example of transcriptional regulation of proteins in response to oxidative stress is the activation of the transcription factor Yap1 in *S. cerevisiae*. Yap1 (Yeast AP-1) is a protein that belongs to the bZIP family of transcriptional regulators and was initially found to be essential in the response of yeast cells to oxidants (including H_2_O_2_ and diamide), as well as certain heavy metals such as cadmium (Schnell and Entian 1991; Kuge and Jones 1994; Wu and Moye-Rowley 1994). Through GFP-tagging of the Yap1 protein, it has been shown that, under normal conditions, the protein localises in the cytoplasm. However, upon treatment of the yeast cells with diamide, the protein rapidly accumulates in the nucleus where it activates a number of genes involved in the oxidative stress response (Kuge et al. 1997). This accumulation in the nucleus under oxidative stress conditions was found to be dependent on the redox regulation of the nuclear export signal (NES) of Yap1. More specifically, redox signals are able to block the export of Yap1 from the nucleus through the modification of the NES (Kuge et al. 1997, 1998; Delaunay et al. 2000). What is particularly interesting in this case is that the modification of the NES differs depending on whether the response was caused by H_2_O_2_ or by diamide. In the case of elevated levels of H_2_O_2_, it is the formation of an intramolecular disulfide bond between C303 and C598, which masks the NES of Yap1 (Delaunay et al. 2000, 2002). When the cells are stressed with diamide, we have the formation of different disulfide bonds within the C-terminal cysteine-rich domain (c-CRD) (C598, C620 and C629), which again leads to a masking of the NES of the protein and allows for its retention in the nucleus (Kuge et al. 2001).

The activation of Yap1 by H_2_O_2_ is not a direct process. Instead, the presence of an additional protein, Gpx3, is required (Fig. 3). Glutathione peroxidase 3 (Gpx3), also known as Hyr1 (hydroperoxide resistance 1) or Orp1 (oxygen receptor peroxidase 1) (Delaunay et al. 2002), acts as the sensor of the levels of H_2_O_2_ within the cell and is the protein responsible for the introduction of the intramolecular disulfide bond of Yap1. Upon exposure to H_2_O_2_, Gpx3 C36 becomes sulfenylated and is able to form a transient mixed disulfide intermediate with Yap1 C598. The formation of this intermediate is essential for the oxidation and subsequent activation of Yap1 (Delaunay et al. 2002; Paulsen and Carroll 2009).

On the other hand, the post-translational modification of proteins is another way through which cells can respond to oxidative stress, which is true of the oxidative inhibition of proteins involved in glycolysis (Grant 2008). Under specific oxidative stress-inducing circumstances, the cells are able to inhibit a series of glycolytic enzymes, including glyceraldehyde 3-phosphate dehydrogenase (GAPDH), which, in turn, inhibit glycolysis and lead to the activation of the pentose phosphate pathway (Ralser et al. 2007). Under these conditions, glucose 6-phosphate dehydrogenase (G6PDH) and 6-phosphogluconate dehydrogenase (6PGDH) are activated, leading to the production of NADPH (Slekar et al. 1996; Morano et al. 2012). The generation of NADPH through this process is especially important, because it acts as the main source of reducing potential for most redox regulatory enzymes, including the two main pathways that control the cellular redox homeostasis: the thioredoxin and glutaredoxin systems (Grant 2008; Morano et al. 2012).

Thioredoxins and glutaredoxins are small oxidoreductases, with conserved structural similarity, particularly in the region of their active sites (Fig. 3). These active sites are characterised by the presence of two conserved cysteine residues, which are indispensable for the function of these proteins. They are thought to play an important role in several cellular processes, including protein folding and repair. Despite their functional similarities, these two classes of proteins differ in the way in which they are regulated: the inactive disulfide-bonded forms of thioredoxins are recycled through the function of thioredoxin reductases and NADPH, while glutaredoxins are recycled by NADPH indirectly, through the transfer of electrons to GSH via the activity of glutathione reductases (Glr) (Holmgren 1989; Trotter and Grant 2002; Wheeler and Grant 2004).

The yeast *S. cerevisiae* contains 3 thioredoxins (Trx1, Trx2, Trx3) and 2 thioredoxin reductases (Trr1, Trr2). Of these proteins, Trx1, Trx2 and Trr1 comprise the cytosolic thioredoxin pathway, while Trx3 and Trr2 make up a complete thioredoxin pathway which is resident within the mitochondrial matrix (Pedrajas et al. 1999; Miranda-Vizuete et al. 2000; Trotter and Grant 2005). The latter is thought to function in order to protect the cell against the oxidative stress generated during respiration (Pedrajas et al. 1999; Greetham et al. 2013). The redox states of the two distinct thioredoxin pathways were found to be maintained independently of one another, while cells that were lacking both systems were viable (Trotter and Grant 2005).

Yeast also contains 8 glutaredoxins (Grx1-8), as well as a single glutathione reductase (Glr1). The cytosolic Grx1 and Grx2 are the most well characterised concerning their role in the oxidative stress response and have been found to be dispensable under normal aerobic growth conditions (Luikenhuis et al. 1998). Grx3 and Grx4 have been found to localise in the nucleus and seem to play an important role in the intracellular trafficking of iron (Mühlenhoff et al. 2010). Grx5 is also associated with cellular iron metabolism and, more specifically, the biogenesis of mitochondrial [4Fe-4S] cluster assembly in the mitochondrial matrix (Rodríguez-Manzaneque et al. 2002). Grx6 and Grx7 have not been extensively characterised, but are resident in the ER and Golgi and are thought to play a role in the regulation of the oxidative state of sulfhydryls in the early secretory pathway (Mesecke et al. 2008; Izquierdo et al. 2008). Grx8 was identified as another glutaredoxin-like protein (Mesecke et al. 2008), but a study of this protein showed that it is unlikely to act as an oxidative stress defence mechanism (Eckers et al. 2009).

In addition to the two systems described above, yeast cells also contain a series of other mechanisms to combat the ROS that are produced, including catalases and superoxide dismutases. The former play a role in the cellular response to H_2_O_2_ and can be found in the peroxisomal and mitochondrial matrices (Cta1) and the cytosol (Ctt1). The latter are involved in the detoxification of O_2_^−^ and can be found in the cytosol and IMS (Sod1), as well as in the mitochondrial matrix (Sod2) (Morano et al. 2012).

## Disulfide bond formation in the bacterial periplasm, the ER and the mitochondrial IMS

**Fig. 4:**
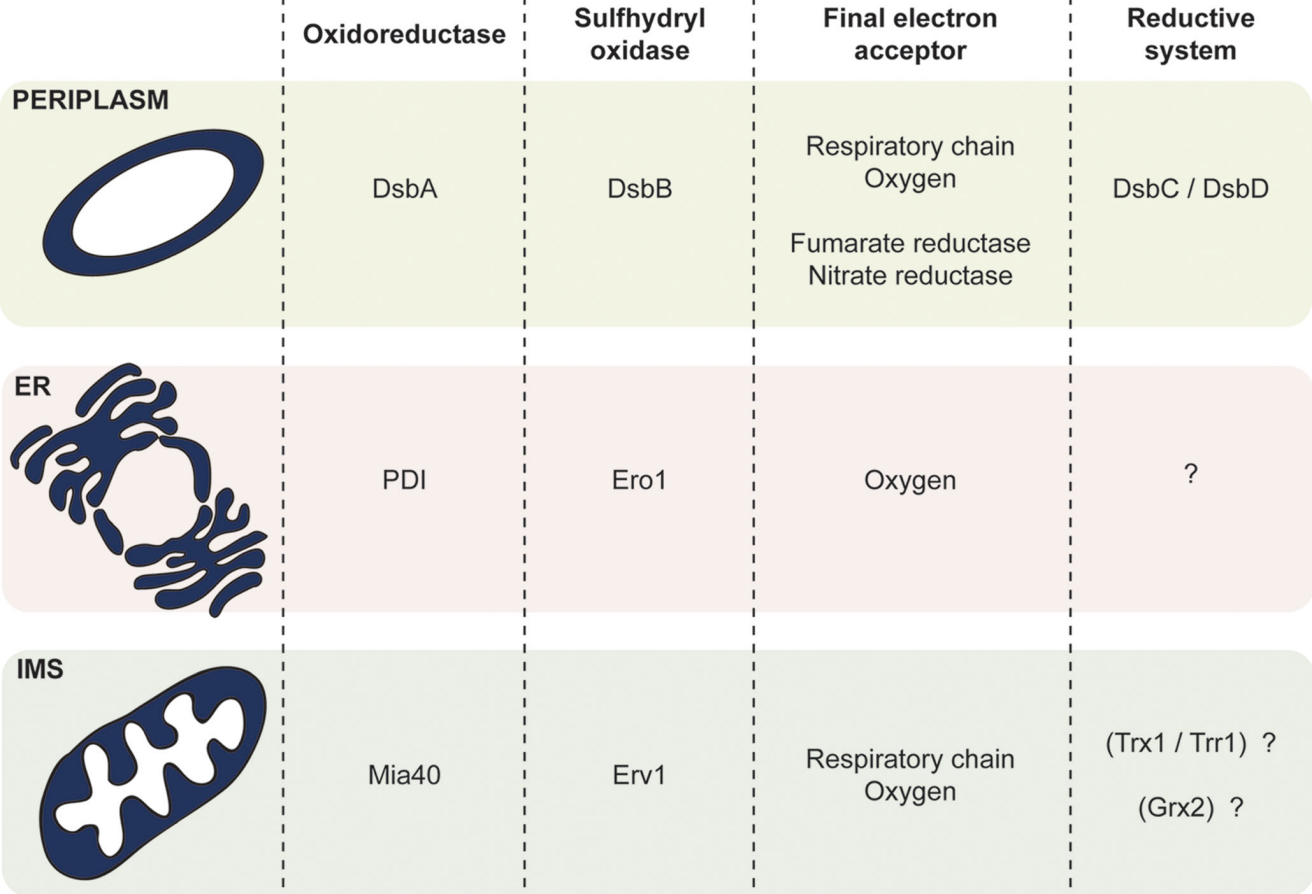
Summary of the components for disulfide bond formation in the bacterial periplasm, the endoplasmic reticulum (*ER*) and the mitochondrial intermembrane space (*IMS*). Each of the compartments where oxidative folding occurs is highlighted in *dark blue*. All three compartments have a similar layout and contain proteins with comparable functions. The main difference is present in the *last column*. The only compartment with a well-characterised reductive system is the periplasm. The ER has no known reductive pathway. In the IMS, the recent localisation of Grx2 and Trx1/Trr1 gives rise to a series of new questions concerning the characterization of a reductive pathway in this particular compartment

The process for the formation of disulfide bonds in cysteine-containing proteins has been extensively described for the cellular compartments that are known to allow the formation of these covalent bonds: the periplasm of bacteria, the endoplasmic reticulum (ER) and, most recently, the mitochondrial IMS (Fig. 4). What is particularly interesting about these three compartments is that they are separate from the site of translation (cytosol) and contain a series of different chaperones, through which the folding and formation of disulfide bonds can occur. The proteins that are targeted to these three compartments are synthesised in a reducing environment, which contains machineries, such as the thioredoxin and glutaredoxin systems, to block the formation of disulfide bonds and keep the proteins reduced (Herrmann and Riemer 2014). Once the proteins have reached their final destination, they interact with the resident chaperones of each compartment, are recognised by the relevant organellar oxidative folding machinery and thus obtain their final, folded form.

### Regulation in the bacterial periplasm

Proteins that are targeted to the periplasm of bacterial cells are synthesised in the cytosol and become transported across the cytoplasmic membrane either through the classical secretory (*Sec*) pathway (Collinson et al. 2015) or through the twin-arginine translocation (*Tat*) pathway (Palmer and Berks 2012). Specifically, in *Escherichia coli*, the vast majority of unfolded proteins become secreted into the periplasm through the *Sec* pathway, while a much smaller number of proteins are dependent on the *Tat* pathway and are transported across the membrane in a folded state (Merdanovic et al. 2011).

As the proteins are secreted through the *Sec* translocase in an unfolded state, they have to fold after targeting to the periplasm. This folding process cannot occur in the same way as it does in the cytosol, due to the lack in the periplasm of ATP and ATP-dependent chaperones like the members of the Hsp60, Hsp70 and Hsp90 families (Herrmann and Riemer 2014).

The periplasm itself is a highly changeable environment due to the presence of porins in the outer membrane and the direct exposure to the ever-changing extracellular environment. Due to the absence of classical chaperones that could help maintain the correct structure of the periplasmic proteins, the majority of proteins in this compartment utilise another protein-stabilising mechanism, namely the introduction of disulfide bonds, to retain their folding and functionality.

The system responsible for the introduction of disulfide bonds in the bacterial periplasm is the Dsb system (disulfide bond formation). The two main components of the Dsb system are the oxidoreductase DsbA and the sulfhydryl oxidase DsbB (Bardwell et al. 1991, 1993). DsbA contains a catalytically active CX2C motif, which, when oxidised, is able to catalyse disulfide bond formation. During this process, the disulfide bond in the DsbA active site is transferred to the substrate through the formation of a transient mixed disulfide intermediate. As a result, DsbA itself becomes reduced and is recycled through the function of DsbB. In a similar manner to DsbA, DsbB also contains a cysteine pair within its active site, which, when oxidised, is able to transfer the disulfide bond to DsbA. DsbB itself becomes re-oxidised via a charge transfer with the quinone co-factor and the electrons are shuttled via this co-factor to the respiratory chain or other terminal oxidases (Bader et al. 1999; Kadokura and Beckwith 2010). The introduction of disulfide bonds in this manner is a process that can occur either co- or post-translationally.

In parallel to the DsbA/DsbB system, there are two other components of the Dsb system, DsbC and DsbD, that constitute the reductive/isomerisation branch of the pathway and operate in the same compartment. These proteins are involved in the recognition and correction of non-native disulfides and are especially important as they provide a critical quality control activity during the oxidative folding process (Shevchik et al. 1994; Missiakas et al. 1995). Such a mechanism is essential because non-native disulfides can form relatively easily, when two cysteine residues that are not normally present as disulfide-bonded come into proximity of one another during the folding process and become oxidised.

The recognition of the wrongly-folded protein occurs via a large partially hydrophobic pocket that is formed through homo-dimerisation of DsbC. This brings the substrate into proximity with the active CX2C motif of DsbC, which interacts with the non-native disulfide and removes it. This process can only occur if the active site cysteine motif of DsbC is present in a reduced state. This is maintained through the function of a fourth protein, DsbD. DsbD itself is membrane-bound, with two domains (α and γ) soluble in the periplasm and one domain (β) anchored within the cytoplasmic membrane. The maintenance of DsbD in a reduced—and thus functional—state occurs through a continuous electron flow that is dependent on the cysteine residues in the three domains of DsbD. More specifically, the bacterial cytoplasmic thioredoxin system is responsible for the reduction of the cysteine residues in the β-domain and, from then on, the electrons flow to the catalytic cysteine residues in the α-domain via the ones that are present within the γ-domain (Collet et al. 2002).

### Regulation in the endoplasmic reticulum (ER)

Just as in the case of the bacterial periplasm, proteins that are destined for the ER are synthesised in the cytosol and are transported through the ER membrane via the *Sec* translocon. During translocation, as well as after complete entry into the ER, they interact with a number of different chaperones and folding factors that enable them to become properly folded and functional (e.g. members of the Hsp70 and Hsp90 families) (Anelli and Sitia 2008). Alternatively, the correct folding of cysteine-containing proteins is achieved though the function of protein disulfide isomerases (PDIs). PDIs are oxidoreductases that contain thioredoxin-like domains with characteristic CX2C motifs in their active sites. There are 5 members of the PDI family in yeast and 20 in mammals, with the most well characterised being the mammalian PDI (Hatahet and Ruddock 2009; Benham 2012).

During the oxidation reaction, PDI acts as an electron acceptor in the thiol–disulfide exchange reaction and this process leads to the introduction of a disulfide bond in the substrate (Oka and Bulleid 2013). From PDI, the electrons are then transferred to the FAD-containing sulfhydryl oxidase Ero1 (ER Oxidoreductin 1) and, finally, to molecular oxygen, in a process that is dependent on the cysteine residues of Ero1 (Gross et al. 2006; Baker et al. 2008).

The oxidative folding pathway in the ER is not without fault and, as such, it is possible that non-native disulfide bonds will also be formed, just as happens in the bacterial periplasm. This is where the mechanism differs quite significantly from the periplasmic one. PDI can actually catalyse isomerisation of non-native to native disulfides, as it can act as an electron donor and reduce the non-native disulfide bonds (Oka and Bulleid 2013). This is a process that can be performed by certain members of the PDI family. A good example of one such member is ERdj5 (endoplasmic reticulum DnaJ homology 5), which contains a thioredoxin-like domain with a relatively low reduction potential, thus making it a better reductase than oxidase (Bulleid and van Lith 2014).

There has been extensive work in recent years in order to understand the mechanisms through which disulfide bonds can become reduced in the ER. However, such mechanisms have remained elusive and we can only speculate on what may be happening. The two most prevalent hypotheses are based on the oxidation of NADPH by either a glutathione or thioredoxin reductase.

The first hypothesis assumes the existence of an ER-localised glutathione reductase that could reduce the GSSG found in the ER. However, as no such protein has yet been found, it has also been hypothesised that the regulation of GSH levels in the ER could also be achieved through the transport of GSH and GSSG between the cytosol and the ER, with the reduction of GSSG happening in the cytosol. This seems more unlikely, due to the fact that the ER membrane seems to be impermeable to GSSG (Bánhegyi et al. 1999; Bulleid and van Lith 2014).

The second hypothesis is also based on the idea that PDI can be recycled through the function of either an as yet unknown ER-localised thioredoxin reductase or the existing cytosolic one. In the case of a putative ER-localised protein, the reduction process would be relatively simple, with the thioredoxin reductase utilising NADPH to directly reduce PDI. On the other hand, it has been hypothesised that an electron shuttle similar to the one operating in the bacterial periplasm could be acting to transfer electrons from reduced thioredoxin in the cytosol to a membrane-anchored protein similar to DsbD and, finally, to PDI (Bulleid and van Lith 2014).

### Regulation in the mitochondrial IMS

The third cellular compartment known to allow the formation of disulfide bonds in cysteine-containing proteins is the mitochondrial IMS. As the IMS has a rather restricted volume, it was initially thought that this compartment only contained around 12 proteins (Martin et al. 1998). However, recent detailed analyses of the yeast *S. cerevisiae* IMS proteome using high-resolution mass spectrometry identified 51 proteins in this compartment, most of which have also been verified biochemically (Vögtle et al. 2012). Another study, in which the authors have attempted to characterise the human IMS proteome using ratiometric APEX tagging, was able to identify 127 IMS proteins, including 16 proteins that had not previously been found to localise to mitochondria (Hung et al. 2014).

Just like the bacterial periplasm, the IMS is devoid of any proteins of the major ATP-dependent chaperone families (e.g. Hsp60, Hsp70, Hsp90). However, the IMS houses the ATP-independent small TIM chaperone system that allows the targeting and insertion of OM and IM membrane proteins (Petrakis et al. 2009). This system is functionally equivalent to the periplasmic membrane protein chaperones SurA and Fkp (Alcock et al. 2008) and, although the proteins have no homology at the level of their amino acid sequence, they share a common substrate binding recognition pattern. Additionally, there are cases of very specialised chaperone proteins, such as Ccs1 (Suzuki et al. 2013; Varabyova et al. 2013) which is thought to interact specifically with Sod1 and drive its import into the IMS, as well as the heme lyases, which play a role in the import of cytochrome *c* (Nargang et al. 1988).

Another factor that seems to play a role in the folding of protein in the IMS is the AAA protease Yme1 (Schreiner et al. 2012). This protein is anchored to the inner mitochondrial membrane and its functional domains are exposed to the IMS. It has been shown that Yme1 plays an important role in the folding and prevention of aggregation of IMS proteins, such as Cox2 (Fiumera et al. 2009). Cells that lack Yme1 display abnormal mitochondrial morphology (Campbell and Thorsness 1998), which could be explained by the aggregation of the components of the MICOS complex (Schreiner et al. 2012).

The system responsible for the introduction of disulfide bonds in the IMS is the MIA machinery. As explained above, the two main components of this machinery are the essential proteins Mia40 and Erv1, which have distinct roles. Mia40 is the oxidoreductase responsible for the introduction of the disulfide bonds into the substrate proteins (Terziyska et al. 2009; Tienson et al. 2009; Banci et al. 2009), while the sulfhydryl oxidase Erv1 is, in turn, responsible for the recycling of Mia40 to its active, oxidised state (Baker et al. 2008; Ang and Lu 2009; Lionaki et al. 2010).

Mia40, although functionally equivalent to the two oxidoreductases analysed earlier (DsbA in bacteria and PDI in the ER), does not contain any thioredoxin-like fold, which is a salient feature of the bacterial and ER systems. Instead, its structure is characterised by two important structural elements: a redox active CPC motif, which can readily switch between an oxidised and a reduced state, and which is positioned directly on top of a shallow hydrophobic cleft where binding of the substrates occurs (Banci et al. 2010). Substrate proteins are able to interact with Mia40 through binding at the hydrophobic cleft. These two structural elements underpin a two-step ‘sliding docking’ mechanism that has been proposed to account for the oxidative folding process (Sideris et al. 2009; Banci et al. 2010). In the first step (‘sliding’), the substrates are accommodated by the binding cleft via non-covalent, primarily hydrophobic interactions engaging the hydrophobic targeting signal of these preproteins (Sideris et al. 2009; Milenkovic et al. 2009; Longen et al. 2009; Sideris and Tokatlidis 2010). This first step allows the cysteine residues of the substrate protein to come into close proximity and directly juxtapose to the oxidised cysteines of the CPC motif of Mia40. In the second step (‘docking’), a transient disulfide intermediate is formed between the second Cys of the CPC motif of Mia40 and the docking Cys of the substrate. A nucleophilic attack by the partner substrate cysteine creates the intramolecular disulfide bond on the substrate, thus leaving the Mia40 CPC motif in a reduced state. Mia40 thereby transfers its disulfide to the substrate protein, assisting its folding and consequent retention within the IMS (Sideris and Tokatlidis 2010; Banci et al. 2010). As Mia40 itself becomes reduced in this process, it requires re-oxidation of the CPC motif in order to become functional again. This is the role that is fulfilled by Erv1. Just like DsbB and Ero1 (in the periplasm and ER, respectively), Erv1 binds FAD, to which it shuttles the electrons that arise from the interaction of the redox active CX2C motif at the N-terminus of Erv1 with Mia40, a process which leads to the re-oxidation of Mia40 (Lange et al. 2001). Once the electrons are in the FAD domain of Erv1, they are then transferred to molecular oxygen either directly or via cytochrome c and cytochrome c oxidase (Farrell and Thorpe 2005).

Contrary to PDI, Mia40 has not been shown to have any isomerisation activity in vivo. There have been studies that show an interaction between glutathione and Mia40, which leads to a semi-oxidised redox state of Mia40. As this is reminiscent of the redox state of PDI family members in the ER, it was thought that Mia40, in its reduced state, might be able to act as a reducing oxidoreductase (Riemer et al. 2009). The only evidence that Mia40 may act as a reductase comes from an in vitro reconstitution setup, in which Mia40 was shown to have the capacity to reduce the substrate protein Cox17, albeit at a much poorer rate than PDI or DsbA (Koch and Schmid 2014a, b).

It is not actually known what happens to Mia40 substrates with incorrectly formed disulfide bonds. Until now, there had been no proof that a reductive system works within the IMS. However, two recent publications have identified the putative presence of two known cytosolic reductive pathways in the IMS: the thioredoxin pathway and the glutaredoxin pathway (Vögtle et al. 2012; Kojer et al. 2015).

In the updated yeast IMS proteome, Vögtle and coworkers were able to identify the presence of Trx1 and its partner Trr1 in the IMS (Vögtle et al. 2012). The discovery of a complete thioredoxin pathway in this particular compartment is interesting as they are proteins that could play an important role in the redox regulation of the IMS. The thioredoxin pathway has been very well described in the cytosol and plays an important role in the maintenance of proteins in a reduced, non-disulfide bonded state. Oxidised substrates are recognised by Trx1 and, through the formation of a mixed disulfide intermediate, Trx1 itself becomes oxidised. Trx1 is recycled by interacting with Trr1 and Trr1 itself becomes reduced by utilising electrons from NADPH (Trotter and Grant 2005). Thus, it is possible that, in the IMS, the two proteins are involved in the recognition and breakage of incorrect disulfide bonds and could work together with the components of the MIA machinery in order to ascertain the correct folding of the cysteine containing proteins of this particular compartment.

A recent study by the Riemer group showed that the IMS seems to harbour glutaredoxin activity, carried out mainly by Grx2 (Kojer et al. 2015). Grx2 is a dually localised protein, which is produced in two forms: a shorter form which is localised in the cytosol, and a more elongated form which is targeted to the mitochondrial matrix (Porras et al. 2006, 2010). The authors suggested that a small fraction of the shorter cytosolic form of Grx2 is imported into the IMS and is able to influence the redox state of IMS proteins through the control of the glutathione pool of this particular compartment. They found that altering the levels of Grx2 in the IMS leads to a shift in the redox state of Mia40, making it present in a primarily reduced state, thus indirectly affecting the import and folding of Mia40-dependent substrates (e.g. Atp23 and Ccs1) (Kojer et al. 2015). Interestingly, Grx2 was not identified in the yeast IMS proteome study (Vögtle et al. 2012), a fact that can possibly be attributed to the very low levels present in the IMS, as hypothesised by Kojer et al. (2015).

No homologues for Trx1, Trr1, Gpx3 and Grx2 were detected in the human IMS proteome, but what is quite intriguing is that, within this list, we can find proteins such as the peroxiredoxins PRDX3 and PRDX4, as well as the thioredoxin-domain containing protein TXNDC12, which hint at the putative presence of a reductive mechanism in the human mitochondrial IMS (Hung et al. 2014).

## Perspectives

The great number of mitochondrial protein import components raises important questions about their evolutionary conservation. The majority of the fundamental molecular machines that ensure correct targeting and sorting to the organelle are conserved among higher eukaryotes. However, a combination of bioinformatics analyses and biochemical characterisation in different species revealed that the mitochondrial protein import machines either have identifiable ancestral homologues in the bacterial endosymbiont or have derived in evolution independently from a bacterial origin. An example of the former is the porin-like Tom40 channel and of the latter the family of small Tim proteins. A detailed description of the evolutionary origin has been discussed in recent excellent reviews (Hewitt et al. 2014).

In this review, we have focused on the mechanistic and regulatory aspects of the protein biogenesis system in the IMS. Given the links of this system to redox regulation and possibly other regulatory cues in the cell, it is apparent that dysregulation of mitochondrial protein biogenesis will have links to the physiology of the cell. One of the future challenges in the field will be to establish these links in human cells and animal disease models of neurodegeneration, cancer and diabetes in which mitochondrial homeostasis is of paramount importance.

The protein import process into mitochondria has been thought for a long time to be a constitutive process. Recent work, however, has demonstrated that this is not the case and that phosphorylation modulates mitochondrial import at both the level of import components and some preproteins themselves (Schmidt et al. 2011; Rao et al. 2011). It is currently unclear whether other types of post-translational modifications, such as thiol modifications, may also play a similar regulatory role. The presence of the redox-regulated protein import and folding process involving the MIA pathway puts the IMS at centre stage in the network of redox homeostasis in cells. The coordination of the oxidative branch with a putative reductive branch of the pathway is critical for quality control and maintaining the redox balance in this compartment. Clearly, further work is needed to address the mechanistic details of these processes, and will require the identification of all the important players (small redox active molecules and proteins) and their interactions. In this respect, it will be of particular interest to dissect the changes induced on the IMS proteome in response to different types of oxidative and reductive cellular stress. As our understanding of the ramifications of the redox-regulated oxidative import and folding in the IMS matures, it will be exciting to discover new pathways for the targeting of important molecular players to this compartment.
